# Author Correction: Analytical investigations of propagation of ultra-broad nonparaxial pulses in a birefringent optical waveguide by three computational ideas

**DOI:** 10.1038/s41598-024-61202-3

**Published:** 2024-05-14

**Authors:** Yuanyuan Liu, Jalil Manafian, Gurpreet Singh, Naief Alabed Alkader, Kottakkaran Sooppy Nisar

**Affiliations:** 1 Department of Mathematics and Artificial Intelligence, Lyuliang University, Lüliang, China; 2 Department of Applied Mathematics, Faculty of Mathematical Sciences, University of Tabriz, Tabriz, Iran; 3 Natural Sciences Faculty, Lankaran State University, 50, H. Aslanov str., Lankaran, Azerbaijan; 4 Department of Applied Sciences, Chitkara university institute of Engineering and Technology, Chitkara University, Punjab Rajpura, India; 5 Department of Sustainable Finance, Plekhanov Russian University of Economics, 117997 Moscow, Russia; 6 Department of Mathematics, College of Science and Humanities in Alkharj, Prince Sattam Bin Abdulaziz University, Alkharj, 11942 Saudi Arabia

Correction to:* Scientific Reports* 10.1038/s41598-024-56719-6, published online 15 March 2024

The original version of this Article contained an error in Affiliation 6, which was incorrectly given as ‘Department of Mathematics, College of Arts and Sciences, Prince Sattam bin Abdulaziz University, Wadi Aldawaser, 11991, Saudi Arabia’. The correct affiliation is listed below:

Department of Mathematics, College of Science and Humanities in Alkharj, Prince Sattam Bin Abdulaziz University, Alkharj, 11942, Saudi Arabia

The original Article has been corrected.

